# How do new mothers describe their postpartum sexual quality of life? a qualitative study

**DOI:** 10.1186/s12905-023-02619-2

**Published:** 2023-09-09

**Authors:** Azam Rahmani, Arezoo Fallahi, Leila Allahqoli, Susanne Grylka-Baeschlin, Ibrahim Alkatout

**Affiliations:** 1 Nursing and Midwifery Care Research Centre, School of Nursing and Midwifery, Tehran University of Medical Sciences, Tehran, Iran; 2 Department of Public Health, Faculty of Health, Kurdistan University of Medical Sciences, Sanandaj, Iran; 3 Midwifery Department, Ministry of Health and Medical Education, Tehran, Iran; 4 Research Institute of Midwifery and Reproductive Health, Zurich University of Applied Sciences, Winterthur, Switzerland; 5 University Hospitals Schleswig-Holstein, Campus Kiel, School of Gynaecological Endoscopy, Arnold-Heller-Str. 3, Haus 24, 24105 Kiel, Germany

**Keywords:** Postpartum sexual quality of life, Qualitative study, Iran, Switzerland

## Abstract

**Background:**

Sexuality plays a critical role in a woman’s postpartum quality of life and also has a strong impact on the quality of her relationship. Given the sparse body of published literature on the subject, we aimed to explore how new mothers explain their postpartum sexual quality of life.

**Method:**

A qualitative study was carried out in Iran and Switzerland from December 2018 to March 2019. Focus groups and semi-structured in-depth interviews (IDIs) were conducted with mothers in the first four months after parturition. Mothers who were older than 18 years, were married or in a stable relationship, and experienced a low-risk vaginal birth or cesarean section participated in the study. We used Graneheim and Lundman’s approach for analyzing the data. Multiple data collection methods, maximum variation sampling, and peer checks were applied to enhance the rigor of the data.

**Results:**

We achieved data saturation after two focus group discussions (FGDs), 15 IDIs in Iran, and 13 IDIs in Switzerland. We extracted three themes for postpartum sexual quality of life: (a) sexual worldview, (b) interpersonal relationship, and (c) postpartum sex storm. The participants described sexual worldview as “sexual beliefs”, “sexual perceptions”, and “sexual behaviors”. The interpersonal relationship consists of “changes in postpartum interpersonal relationships” and “supportive role of the husbands/partners”. The last one, postpartum sex storm, has three categories including “direct changes in sexual life”, “indirect changes in sexual life”, and “resumption of sexual intercourse”. Differences between the two cultures were identified in some subcategories such as “sexual interests”, “comparable to the first intercourse in life”, “negative sexual behaviors of husbands/partners”, “positive sexual behavior of mothers”, “helping with child care and housework”, and “emotional support”.

**Conclusion:**

New mothers explained postpartum sexual quality of life as a three-theme phenomenon. Although most results were similar in both cultures, there were some differences. Our study’s results suggest that sexual quality of life is a topic that encompasses international as well as cultural aspects.

## Background

Sexuality is an important aspect of the quality of life for both men and women [1–3]. Although sexuality is an essential element of postpartum quality of life, it is frequently impaired by the adaptation to the new role of parents [4, 5]. Sexuality in the postpartum period is crucial because it could affect a couple’s interpersonal relationship. Therefore, the restoration of a satisfactory sexual relationship in the postpartum period is very important [6].

New parents experience sexual problems. Depending on how much time has passed since birth, 40% to more than 80% of them experience at least one episode of sexual problem [7–10]. Women after childbirth are confronted with a reduced frequency of sexual activity, low desire for it, dyspareunia, lack of lubrication, and dissatisfaction with sexual life [5, 7, 11, 12]. Several factors have been found to be associated with an impaired sexual life after childbirth, such as higher maternal age, higher educational level and family income, partnership issues, maternal mental health problems, first child, shorter time since childbirth, vaginal births with episiotomies, instrumental births, cesarean section, and exclusive breastfeeding [5, 7, 11, 13–17].

Although many studies conducted around the world have focused on factors related to postpartum sexual health, no study has focused on “postpartum sexual quality of life”. Lidia Pardell-Dominguez et al. (2021) conducted a phenomenological study on “the meaning of postpartum sexual health for women living in Spain”, which reported five themes including “not feeling ready”, “inhibiting factors”, “new reality at home”, “socio-cultural factors”, and “the clinician within the health system” [18].

A directed qualitative content analysis was conducted in Iran (2022) on women’s perceptions and experiences of the concept of postpartum sexual function. Eleven themes emerged from the analysis. Six categories were related to the predetermined components, and five themes were new [19]. A systematic review in 2015 entitled “Postpartum sexual health: a principle-based concept analysis” concluded that postpartum sexual health is conceptually immature and of limited applicability in current maternity care [20]. Since the concept of “sexual quality of life” could differ from “sexual health” and “sexual function” [21–23], and no study has been specifically focused on “postpartum sexual quality of life”, we decided to focus on this issue.

Sexuality is a context-based concept and could differ from one culture to another [24]. We included both extremes; Iran as a developing country and Switzerland as a developed country to identify similarities and differences between the two contexts. In addition, sexuality is a very religious-dependent topic, and these two cultures have different religious beliefs; as some types of sexual behaviors such as premarital sex and masturbation are forbidden religiously and socially in the context of Iran [25]. Also, some norms such as patriarchal sexual behaviors in couples are bold in the Iranian context [26].

Since sexuality plays a critical role in women’s quality of life and due to reduced frequency of sexual activity, low desire for it, dyspareunia, lack of lubrication, exhaustion, insomnia, and fear, the postpartum period is a fragile window in a sexual relationship; moreover because the postpartum sexual quality of life is a subjective concept that could be explored using a qualitative approach; and finally since there was no study in this field, we planned this study to explore how new mothers explain their postpartum sexual quality of life. By explaining postpartum sexual quality of life and the challenges that new mothers experience in two different contexts, we hope to provide new insights into highlighting the importance of and normalizing changes in this period.

## Method

### Design

The present study was part of a joint research project between Switzerland and Iran, which was funded by a Seed Money grant in 2018. A qualitative approach using conventional content analysis was applied because there were no preconceived hypotheses in the field of postpartum sexual quality of life [27]. The study was conducted from December 2018 to March 2019.

### Participant*s*

The study participants were recruited through healthcare centers in Iran and through semi-structured in-depth interviews (IDIs) in Switzerland, which provided postpartum care. Mothers were included if they were primiparous, 18 years or older, married (Iran) or living with a partner as cohabitants (Switzerland), of Iranian nationality (Iran) or spoke German fluently (Switzerland), had experienced a healthy pregnancy, and had a low-risk vaginal birth or a cesarean section without complications during the last four months. We excluded participants who had experienced a negative event, such as the death of a close relative or friend during the last six months, had a reported history of psychological disorders, or did not feel comfortable talking about sex. Since focus group discussions are an effective means of data collection in sexuality research [28, 29], we asked Iranian participants to state whether they were interested in participating in a focus group discussion (FGD) or an IDI. We chose the period extending to four months after childbirth because studies have shown that sexual dysfunction is prevalent in the postpartum period, especially during the first four months after delivery y [8, 10].

### Procedure

Mothers who had delivered in the last four months were identified from a list at the healthcare centers and were invited to participate in the study. Before starting the IDIs and FGDs, all mothers completed a questionnaire that asked for demographic data, such as age, level of education, occupational status, and mode of delivery.

FGDs and IDIs were conducted face-to-face by a female Ph.D. with a background in nursing and midwifery in Iran, and by a female Ph.D. with a midwifery background and a master’s degree in midwifery in Switzerland. The interviewers were experts in qualitative research or were trained in this field. Interviews were conducted in a secluded room at the healthcare centers or at the participant’s home, and all participants provided informed written consent. A semi-structured guide of open-ended questions was used, based on the WHO definition of sexual health (which includes physical, mental, and social wellbeing in relation to sexuality) and the dimensions of sexual quality of life (which include physical, social, emotional, and psychological aspects) [30]. It covered several dimensions of sexuality, including the physical, social, emotional, mental, and psychological aspects. Some items in the interview guide are listed below:

- How has your sexual life been after giving birth?
- What changes (physical, emotional, and psychological) have you experienced in your life and sexual life after the delivery?
- What factors do you consider important in the quality of your sexual life after childbirth?
- How is your interpersonal relationship with your husband/partner?
- What are the dominant sexual beliefs which govern your sexual relationship?
- What were some of the challenges you confronted in your interpersonal and sexual relationship after giving birth?

The interview guide was available in Farsi and German. The FGDs and IDIs were recorded and transcribed verbatim.

The following strategies enabled us to overcome the language of silence and privacy concerns: (a) controlled glances, gestures, speaking style, and posture, (b) providing a situation for consulting participants on their sexual life after completing the IDIs or FGDs, if needed, (c) ensuring that the results extracted from their private sexual behaviors would only be used for the investigation, (d) dedicating a pseudonym to each participant to ensure their anonymity, and f) moving from non-sexual to sexual items in order to avoid a sense of intrusion beyond personal boundaries.

### Data Analysis

We applied Graneheim and Lundman’s approach for qualitative analysis of data [31]. The data analysis consisted of the following steps: (1) extracting the units of meaning from the statements; (2) conducting line-by-line coding, in which the codes were created on the basis of repeated discussions between the researchers; non-verbal expressions were also coded and included in the data analysis; (3) categorizing similar codes as the same meaning; and (4) developing themes on the basis of codes with similar meanings. Repeated in-depth discussions between the researchers from both countries were held to extract themes. The MAX-QDA 10 and Atlas.ti 8 software were used for data analysis in Iran and Switzerland, respectively.

### Rigor

To ensure the rigor of the study, we requested the services of an expert as a second coder for the qualitative approach (AF). The Iranian coder (AR) also communicated with the Swiss coder (SG-B). We requested five participants to provide a critique of the summarized interviews and findings (member check). The confirmability of the findings was achieved by checking the substantive codes and themes, which were checked by four experts in the qualitative approach of content analysis and sexuality research (peer check). The maximum variation sampling (different socioeconomic status, age, mode of delivery, and education) enhanced the transferability of data.

## Results

Data saturation was achieved after two FGDs and 15 IDIs in Iran, and 13 IDIs in Switzerland. A total of 36 mothers participated in the study, including 23 Iranians and 13 Swiss. The IDIs and FGDs lasted between 14 and 65 min. In Iran, all participants were married and between 19 and 35 years old. Sixteen had a moderate economic status, 14 were housewives, and 13 had caesarean sections. Most participants (n = 9) had a bachelor’s degree. In Switzerland, participants were between 25 and 35 years old. Half of them were married (n = 7), and six lived with their partners in a stable relationship. Seven participants had a bachelor’s degree or higher. Six had a spontaneous vaginal birth, three had a caesarean section, and four had an instrumental vaginal birth. The interviews were conducted between 32 and 104 days after childbirth in both countries. Table 1 summarizes the characteristics of the study participants.

**Table 1:** Characteristics of participants in FDGs and IDIs

		Switzerland (n = 13) N (%)	Iran (n = 23 ) N (%)
Age (years)	< 20	0 (0)	1 (4.3)
	21–30	6 (46.2)	10 (43.5)
	> 30	7 (53.8)	12 (52.2)
Mode of delivery	Natural delivery	10 (76.9)	10 (43.5)
	Cesarean section	3 (23.1)	13 (56.5)
Occupational status	Housewife	0 (0)	14 (60.9)
	Employed	13 (100)	9 (39.1)
Educational level	Under diploma level	0 (0)	0 (0)
	Diploma	6 (46.2)	8 (34.8)
	University	7 (53.8)	15 (56.2)

We extracted three themes for postpartum sexual quality of life, including: (a) sexual worldview, (b) interpersonal relationship, and (c) postpartum sex storm. Participants described sexual worldview as “sexual beliefs,” “sexual perceptions,” and “sexual behaviors.” The interpersonal relationship consisted of “changes in postpartum interpersonal relationships” and “the supportive role of husbands/partners.” The last theme, postpartum sex storm, had three categories, including “direct changes in sexual life,” “indirect changes in sexual life,” and “resumption of sexual intercourse.” Differences between the two cultures were recognized in some subcategories, such as “sexual interests”, “experiences comparable to the first intercourse in life”, “negative sexual behaviors of husbands/partners”, “positive sexual behavior of mothers”, and “emotional support related to child care and household work”. Themes, categories, and subthemes are summarized in Table 2.

**Table 2:** Themes, categories, and sub-categories

Theme	Category	Sub-category
Sexual worldview	Sexual beliefs	The necessity of sex
		Principles of sex
		Benefits of sex
	Sexual perception	**Sexual interests**
		Sexual experiences
		**Comparable to the first intercourse in life**
	Sexual behaviors	**Negative sexual behaviours of husbands/ partners**
		**Positive sexual behaviour of mothers**
Interpersonal relationship	Changes in postpartum interpersonal relationships	Positive changes
		**Negative changes**
	Supportive role of the husbands	**Helping with child care and house works**
		**Emotional support**
Postpartum sex storm	Direct changes	Sexual desire
		Pleasure of sex
		Frequency of sexual relationships
		Pain
		Duration of sex
	Indirect changes	Body images
		Mood stability
	Resumption of sexual intercourse	Reasons for not resuming sexual intercourse
		When to start having sex after giving birth

The bold subcategories show the difference between the two countries

### Sexual worldview

The sexual worldview encompasses mothers’ “sexual beliefs,“ “sexual perceptions,“ and “sexual behaviors.“ While both cultures were similar in terms of sexual beliefs, there were some differences in the other two categories, “sexual perception” and “sexual behaviors.“

#### Sexual beliefs

In terms of sexual beliefs, many participants believed that sexuality is a natural human need and important for life, health, and the relationship with the husband/ partner. Mothers also clearly stated that trust in the relationship is important to feel good and safe during sex.

“*I think sexuality is something human. Everybody does it. Yes, well… I think it’s part of a healthy relationship.“* (IDI in Switzerland, 25 years old, 92 days after spontaneous vaginal birth, medical secretary).

“Sex *is the foundation of married life.“* (IDI with an Iranian participant, 30 years old, 80 days after caesarean, housewife).

#### Sexual perception

The mothers’ perception and experience of sexuality differed widely. While some participants liked variety, others stated that a happy sexual life requires hard work and does not succeed on its own.

*“I like to experience diverse sexual behaviors. I do not like repetitive models of sex that are only done explicitly to satisfy people’s sexual needs*.“ (IDI with an Iranian participant, 32 years old, 62 days after a caesarean birth, employee).

“*And if you’re not happy with something, you can work on it, no matter what it is. It’s a long and hard way. And I think that’s the secret of a happy sex life.“* (IDI in Switzerland, 29 years old, 91 days after a spontaneous vaginal birth, self-employed beautician).

While participants from both countries discussed their positive experiences with sexual interests during the postpartum period, there were cultural differences. Several Iranian mothers mentioned that their husbands suggested anal sex due to dyspareunia, but they did not find non-vaginal sex pleasurable and found anal sex to be particularly painful. On the other hand, some Swiss mothers reported enjoying the use of sex toys, also during this time.

“*If it would be anal sex, I absolutely refuse. Once during my engagement, my husband made such a suggestion, but because it was very painful, he doesn’t suggest it anymore.“* (IDI. Iranian mother, 27 years old, caesarean, 32 days after birth, housewife).

“*We were already very open before. We also experimented and have some toys at home, this hasn’t changed. “(*IDI. mother in Switzerland, 34 years old, 94 days after spontaneous birth, administration assistant).

Swiss mothers drew a comparison between their first sexual encounter after giving birth and their initial experience of intercourse, whereas no such comparison was mentioned by participants from Iranian culture.

“*It is* like *having sex for the first time in your life. It was definitely like this for me. At that time, I also had the feeling that something had happened….“* (IDI. mother in Switzerland, 35 years old, 90 days after instrumental birth, physiotherapist).

“It *was like finding contact again to my body. I found that quite fascinating. Like relaxing again. It was like having sex for the first time (in life) “(*IDI. mother in Switzerland, 28 years old, 98 days after vaginal instrumental birth, social education worker).

#### Sexual behaviors

The sexual behaviors of mothers differed between the two cultures. On the one hand, some Iranian mothers reported negative sexual behaviors from their husbands/partners, such as neglecting their sexual needs and disregarding their readiness to engage in sexual activity. No Swiss mother mentioned being forced to have sex against her will.

“*Sometimes, my husband disregards my desire. For example, if I suggest having sex, he says that I am tired.“* (FGD. Iranian mother, 19 years old, caesarean, 51 days after birth, housewife).

On the other hand, mothers in both cultures displayed positive sexual behaviors, but the specifics differed. Iranian mothers’ positive sexual behaviors included initiating sexual activity, attempting to understand their husbands’/partners’ sexual needs, adapting their approach to sex depending on the circumstances, and agreeing to their husbands’ requests even if they were not enthusiastic about it. However, in Swiss culture, accepting a partner’s request for sex without genuine desire would not be considered a positive behavior and is not culturally expected. Furthermore, only a few Swiss mothers reported having sex without desire.

“*I know what makes him sexually enjoy the most, I do the same for him.“* (IDI. Iranian mother, 27 years old, normal delivery, 87 days after birth, housewife).

“*When I didn’t want to have sex, if my husband wanted it, I would agree to have a relationship*.“ (IDI. Iranian mother, 34 years old, caesarean, 90 days after birth, housewife).

“*Yes, before (giving birth), I often engaged in sex simply because I thought I needed to satisfy him. Not that he would cheat on me, but because he deserves it somehow. He is so nice and kind…. And yes, then I just pretended sometimes (to have an orgasm)*” (IDI. mother in Switzerland, 29 years old, 91 days after spontaneous birth, beautician).

### Interpersonal relationship

Most participants expected their husbands/partners to understand their situation, care about their emotional needs, seek their opinions on life decisions, and cooperate in household chores. The interpersonal relationship consists of two categories: “changes (positive and negative) in postpartum interpersonal relationships” and “supportive role of the husbands/partners.“ The “supportive role of the husbands/partners” category had two subcategories: “helping with child care and housework” and “emotional support.“ The difference between the two cultures was more remarkable in terms of interpersonal relationships, as only one subcategory (positive changes in interpersonal relationship) was the same in both cultures.

#### Changes in postpartum interpersonal relationships

The participants reported many changes in their relationships with their husbands/partners after giving birth to their children. Some changes were positive, and others were negative. Mothers receiving more attention from their husband and an improved relationship were among the positive changes. In some cases, conflicts arose regarding how to care for the baby.

“*My husband has become kinder after the baby came. He always tries to care for me.“* (FGD, Iranian participant, 19 years old, caesarean section, 51 days after birth, housewife).

“*But it’s also an additional factor of dispute because I mean, everybody wants only the best [for the baby] but has maybe a slightly different idea*.“ (Individual interview in Switzerland, 33 years old, 80 days after a caesarean section, research assistant).

The interaction of mothers with their husbands/partners in conflicts includes resolving problems through dialogue, remaining silent during conflicts, and calming down arguments.

“*Any problem we have, we both try not to take it out of the house. We try to solve it ourselves*.“ (Individual interview with an Iranian participant, 27 years old, caesarean section, 75 days after birth, housewife).

“*And there we had a real argument, and then we talked about it.*“ (Individual interview in Switzerland, 29 years old, 98 days after a spontaneous vaginal birth, architect).

In addition to positive behaviors such as resolving conflicts through communication, Iranian mothers also reported negative behaviors such as remaining completely silent during discussions and expressing disapproval through huffing for several days. Meanwhile, Swiss mothers discussed various strategies for managing marital or couple life but also acknowledged that the baby could be an additional source of conflict in the relationship.

“*Because I am a proud person, I don’t go to him after a fight and wait for him to come to me.*“ (IDI. Iranian mother, 31 years old, normal delivery, 81 days after birth, employed).

“*I need to tell him more actively again what bothers me*.“ (IDI. mother in Switzerland, 29 years old, 97 days after spontaneous birth, architect).

“*And we also argue about her (the baby), so sometimes and that’s why it happens …, but yes. So now there is another point of contention*.“ (Ind.-int. mother in Switzerland, 33 years old, 80 days after caesarean section, scientific assistant).

#### Supportive role of the husbands/partners

Most of the participants had positive experiences with their husband’s/partner’s supportive role, but there were also cases of them lacking the opportunity to help due to long working hours. The participants discussed their ideals and expectations regarding their husbands or partners. The majority of participants in both cultures expected their partners to understand their situation, particularly after childbirth, and to provide emotional support. Iranian mothers also expected their husbands/partners to contribute to household affairs. However, some Swiss mothers expressed the expectation that their partners should accept that they are not always the top priority and that they have other responsibilities to attend to, such as caring for the baby.

“*My husband is very busy. He leaves at 7 in the morning and comes home tired at 9 at night. He no longer has the energy to help.“* (IDI. Iranian mother, 31 years old, normal delivery, 71 days after birth, housewife).

“… *but at the beginning of a partnership, a couple is always together, and suddenly the baby is here, and he (the husband) is the second priority. He feels neglected, but he must bear this*.“ (Ind.-int. mother in Switzerland, 33 years old, 80 days after caesarean section, scientific assistant).

Emotional support was another concept that mothers reported. While negative behaviors from husbands or partners were present in both countries, the accounts from Iranian mothers were more severe. Some Iranian mothers reported feeling psychologically abused by their husband’s behavior, including harsh treatment, neglect of their emotional detachment, and failure to meet their expectations. In contrast, no Swiss mothers reported experiencing abusive behavior from their partners. However, a few Swiss mothers expressed feeling overwhelmed by the demands of caring for a baby and a partner simultaneously.

“*I tell my husband that I want to go visit the doctor. I would like you to come with me. He says I have work; I can’t come. My work is more important than anything else.“* (IDI. Iranian mother, 31 years old, caesarean, 78 days after birth, housewife).

“*And then, in the evening, the husband comes home, and he also needs attention.“* (IDI. mother in Switzerland, 29 years old, 97 days after spontaneous birth, architect).

### Postpartum Sex Storm

The last theme, postpartum sex storm, has three categories, including “direct changes in sexual life,“ “indirect changes in sexual life,“ and “resumption of sexual intercourse.“ Both cultures were the same in all categories and sub-categories of this theme.

#### Direct changes in sexual life

After childbirth, couples experienced substantial changes in sexual behavior, describing it as a storm. Many participants reported a decrease in their desire, pleasure, and frequency of sexual intercourse during the postpartum period due to various reasons such as sleep deprivation, fatigue, and less energy. A small percentage of mothers did not experience any changes or reported an improvement in their sexual life.

“*My sexual relationship has deteriorated, and I enjoy it less than before.“* (Individual interview with an Iranian participant, 32 years old, caesarean section, 62 days after birth, employed).

“*I really think my sex has become better; now I enjoy sex more than before.“* (FGD, Iranian participant, 25 years old, normal delivery, 42 days after birth, employed).

“*I think it’s different each time. No two orgasms are the same. But I still experience them, and I don’t think there are extreme differences between now and before.“* (Individual interview in Switzerland, 28 years old, 88 days after an instrumental vaginal birth, social education worker).

#### Indirect changes in sexual life

Participants reported some factors that could affect postpartum sexual quality of life indirectly, such as body image and mood stability. Most participants experienced psychological changes such as stress, anxiety, depression, fear, anger, irritability, and grief in the postpartum period. Some participants said that childbirth had introduced changes such as a greater sense of responsibility, more self-confidence, peace, and hope in their lives.

“*There are moments when I just have to cry. I worry when it’s not going so well especially when she (baby) cries and does not calm down.“* (Individual interview in Switzerland, 32 years old, 104 days after a spontaneous vaginal birth, aspiring secondary school teacher).

“*After giving birth, I think my self-confidence increased because I gradually did everything for the baby and accepted more responsibilities.“* (Individual interview with an Iranian participant, 34 years old, caesarean section, 81 days after birth, employed).

In addition, participants had different feelings, negative and positive, towards their physical changes, such as paying attention to their physical appearance and accepting physical changes.

“*I became obese, and I do not like it… I have to go on a diet.“* (Individual interview with an Iranian participant, 31 years old, caesarean, 84 days after birth, housewife).

“*But otherwise, that a body changes after pregnancy… is also something beautiful. Because it’s a sign that you’re a mommy.*“ (Individual interview in Switzerland, 34 years old, 94 days after a spontaneous vaginal birth, commercial employee).

#### Resumption of sexual intercourse

Some participants stated that they still did not have sex after 40 days or even at the time of the interview at four months after childbirth. The most common reasons were medical advice, lack of a suitable environment, lack of husbands’ requests, and the participants’ excessive fatigue.

“*My husband asked me to have sex several times, but I told him that the doctor has banned sex for three months! Therefore, he did not ask anymore.“* (Individual interview with an Iranian participant, 31 years old, caesarean section, 78 days after birth, housewife).

“*And clearly at the beginning, with these hemorrhoids and so on, it was not possible at all. I was also physically exhausted. I couldn’t have done it at all.*“ (Individual interview in Switzerland, 35 years old, 95 days after a spontaneous vaginal birth, attorney).

**Fig. 1:**
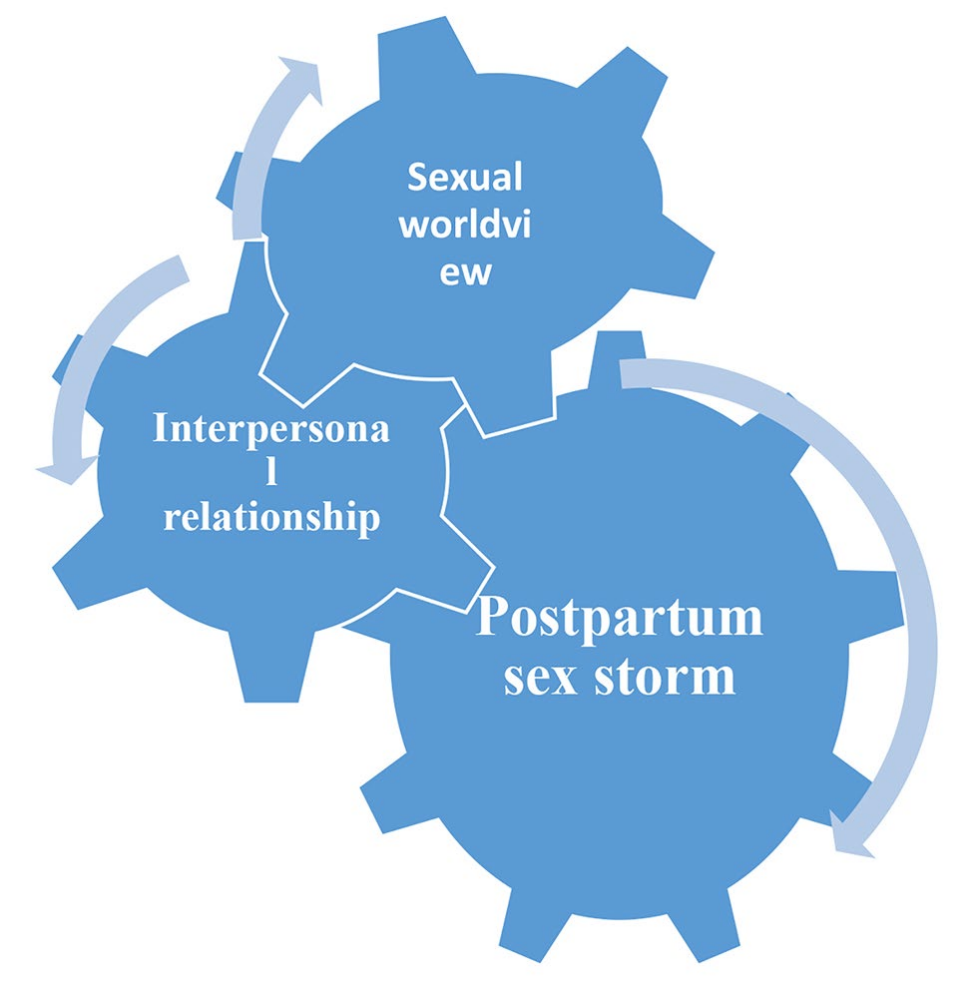
The exemplification of gear wheel of postpartum sexual quality of life

Analyzing the results leads to extracting three domains for postpartum sexual quality of life, including sexual worldview, interpersonal relationship, and postpartum sex storm. According to the participates’ quotes, these three domains interact with each other; if one of them does not work very well, the other domains will not be able to have a suitable performance. Therefore, we exemplified the relationship of these domains as a gear wheel. Figure 1 shows The exemplification of gear wheel of postpartum sexual quality of life.

## Discussion

This is the first study addressing mothers’ experiences in terms of postpartum sexual quality of life. We extracted three domains for postpartum sexual quality of life, including sexual worldview, interpersonal relationship, and postpartum sex storm. The results of a study on “women’s perceptions and experiences of the concept of postpartum sexual function” were similar to ours in some respects and dissimilar in others. This was a directed qualitative study based on the Female Sexual Function Index. Eleven categories emerged from the analysis, six of which were related to the predetermined components of the Female Sexual Function Index, namely sexual desire, arousal, lubrication, pain, orgasm, and satisfaction [19]. However, these categories were not consistent with the results of our study due to the nature of our analysis. Five additional categories that emerged in that study included changes in the frequency of sexual intercourse, disturbed situation, changes in intimacy and relationship, physical (anatomical) changes, and psychological consequences [19]. The new five themes of this study are consistent with the results of our study.

The participants described sexual worldview as their beliefs, perceptions, and behaviors related to sexuality. This theme covers what mothers have felt, believed, and experienced about their sexuality up to this point. The results of a study focused on the “meaning of sexual health in the postpartum period” are partly similar to our results. The sexual worldview described in our study was consistent with a theme conceptualized as “social and cultural backgrounds” in that research. According to that study, women reported certain beliefs and preconceived ideas as being part of their social and cultural backgrounds, which influenced their sexuality after childbirth [32]. In our study, participants also described sexual worldview as their beliefs about sex, including the necessity, principles, and benefits of sex. These beliefs were influenced by their cultural background and shaped their perceptions and experiences of postpartum sexual quality of life. Sexual interests were one of the differences between the two cultures. Iranian mothers expressed a dislike for non-vaginal sex, particularly anal sex. In contrast, some Swiss mothers reported using sex toys, but this was not mentioned by any of the Iranian mothers, since it is not culturally acceptable. While the use of sex toys is becoming more common in other cultures [33], it is considered taboo in Iran. Research indicates that women with diverse sexual orientations, such as lesbian and bisexual, are more likely to use sex toys [34]; however, these orientations are forbidden in Iranian culture. Additionally, the use of sex toys in Iran is often associated with masturbation, which is religiously forbidden.

Another theme extracted in this study is interpersonal relationship. The effects of interpersonal relationships on sexual behaviors are well known [22, 35]. Although a couple’s sexual worldview can influence their sexual experiences, it is often developed and shaped through their interpersonal and sexual relationship with their partner, which is reflected in their sexual script. The sexual script refers to the set of socio-cultural expectations through which individuals learn patterns of “appropriate” sexual desires and behaviors in a specific context [36]. Sexual scripts refer to the cultural expectations and norms that shape people’s beliefs and behaviors related to sexuality. These scripts include both the feelings and behaviors that are considered sexual, as well as those that are deemed acceptable within a particular context or culture [37]. According to the scripting theory, sexual scripts can be divided into three levels: cultural (established regulations and collective implications), interpersonal (communications and behaviors in specific contexts), and intrapsychic (beliefs and desires) [38]. The theme that emerged in our study is directly related to the interpersonal level of the scripting theory, and indirectly to the cultural and intrapsychic levels. Our findings suggest that sexual scripts varied across cultures and stages of life for both Iranian and Swiss participants, and were influenced by other identities such as race, ethnicity, and social class [39]. One of the differences that observed in the two cultures was the management of conflicts. Some Iranian mothers reported that their husbands refused to assist with housework, while this was rarely mentioned in the Swiss context. The reason for this difference may be due to the fact that in Switzerland, men are expected to participate in household chores, although there is still inequality in the distribution of domestic tasks [40]. On the other hand, in Iran, marriage is considered a mutual contract where women are expected to take care of household chores and child-rearing while men are responsible for managing and financing family life.

The last theme that emerged in this study is postpartum sex storm. Participants stated that their personal lives had changed in terms of physical, emotional, and psychological aspects. Other studies have also confirmed these changes, indicating that participants may experience irregular sleep schedules, sleep deprivation, breastfeeding, and physical recovery from childbirth [41, 42]. When women do not get adequate sleep during the postpartum period, they are more likely to experience exhaustion and depression [43], which can be significantly associated with impaired sexual quality of life [41]. Although the postpartum period is primarily a biological experience, it is also a psychological phenomenon that involves significant changes in mood, identity, and lifestyle. In this study, participants reported a variety of psychological changes, such as mood disorders and feelings of sadness, along with adaptations related to becoming a parent and managing the substantial life transition. These changes may involve mood variations, feelings towards the baby and oneself, and adjustments to new roles and responsibilities [44]. The postpartum period is not only a biological and psychological experience but also a social one. Couples must learn how to interact with each other and care for the demands of a new baby during this time. These biological, psychological, and social factors can directly affect postpartum sexual life [41]. Participants in our study reported a decrease in their postpartum sexual desire, pleasure, and frequency of sexual intercourse for various reasons, including sleep deprivation, fatigue, and low energy. In terms of differences between two cultures, in Iranian culture, accepting a husband’s sexual request even if the mother is unwilling to engage in sexual activity is considered a positive behavior. Some Iranian mothers attribute this to their love for their husband. However, in the Swiss context, accepting sexual requests without a genuine desire for sex is rare, as couples usually initiate sexual activity only when both partners are willing. Rejecting sexual demands in Iranian culture is considered unacceptable, and mothers tend to respond positively to their husband’s sexual requests, usually in most cases. This difference can be attributed to cultural and marital expectations in the context of Islamic marriage. According to Islamic teachings, husbands and wives are expected to fulfill each other’s sexual needs [45, 46]. During the interviews, the researcher noticed that some Iranian women consider sex to be a duty rather than a right to be fulfilled. In contrast, in Switzerland, only one woman mentioned that her partner deserved sex, and she agreed to it occasionally despite lacking desire. Most mothers in both cultures expected their husband or partner to wait until they were ready for sexual activity after childbirth. Another finding of this study was related to physical changes after giving birth. Most Swiss mothers found the physical change as a beautiful phenomenon while most Iranian mothers reported that they did not like the obesity that they experienced after delivery. The large number of studies related to body changes and body image after giving birth in the Iranian context [47–52], versus the difficulty in finding such studies in Switzerland may reflect the fact that body image and related concerns in this period are more critical issues for the Iranian mothers. Although Iranian studies have reported different aspects of body image during postpartum, body image dissatisfaction seems a general and universal event after giving birth [53]. While it is supposed that weight gain during pregnancy is not as crucial as other periods during life, studies suggest that new mothers try to meet the standards they had before pregnancy [54]. These standards may be related to emotional and physical challenges that mothers experience in order to return to their pre-pregnancy weight and appearance.

According to the results of our study, some participants were not eager to engage in sexual activity during the postpartum period. Although the published literature has reported inconsistencies in the factors affecting postpartum sexual life, such as the mode of delivery, there is a general agreement that the postpartum period is a time of significant physical, emotional, and psychological changes that can affect sexual desire, pleasure, and frequency of sexual intercourse [55], Several reasons have been cited for a woman’s lack of eagerness to engage in sex during the postpartum period. These reasons may include vaginal pain, fear of rejection by either partner, and body shame [56].

A strength of the present study was its cross-cultural approach, which allowed us to explore similar topics in two different cultures and gain insights into the complexity of women’s sexuality and sexual relationships across different cultural contexts. Sexuality is a topic that encompasses international, cultural, and religious aspects and can vary significantly based on these factors.

However, there were also limitations to this study. One limitation was related to the focus group discussions. Although we initially planned to conduct more than two focus group discussions, we encountered challenges in recruiting participants who were willing to discuss their sex lives with other women. As a result, we expanded our recruitment efforts to include individual interviews, which may have affected the data collection process and the findings of the study.

## Conclusion

New mothers in our study described postpartum sexual quality of life as a three-theme phenomenon that includes sexual worldview, interpersonal relationship, and postpartum sex storm. Understanding the challenges that new mothers face in different contexts can be used to develop educational programs for new couples, which can highlight the importance of and normalize changes during this period and support women in managing these challenges. We recommend providing appropriate and comprehensive resources, such as health promotion strategies, sexual health education, counseling, and other psychosocial interventions, to support women’s postpartum sexual quality of life. We suggest conducting research on new fathers and assessing postpartum sexual life from their perspective.

## Data Availability

The data presented in this study are available on request from the corresponding author.
